# Serum levels of EphA2 are elevated in knee osteoarthritis and associated with disease severity

**DOI:** 10.3389/fmed.2025.1639458

**Published:** 2025-10-08

**Authors:** Maping Xiao, Lei Ji, Shuang Feng, Wenbin Qian

**Affiliations:** ^1^Department of Orthopedics, Nantong Rehabilitation Hospital, Nantong, Jiangsu, China; ^2^Department of Sports Medicine, Nantong Rehabilitation Hospital, Nantong, Jiangsu, China

**Keywords:** osteoarthritis, EphA2, WOMAC, K-L scores I-II, K-L scores III-IV

## Abstract

**Background:**

This study investigated the clinical relevance of serum ephrin type-A receptor 2 (EphA2) in patients with knee osteoarthritis (OA) compared to healthy controls and its association with disease severity, inflammatory markers, oxidative stress, and cartilage metabolism.

**Methods:**

A total of 258 participants, including 138 patients with primary knee OA and 120 age- and sex-matched healthy controls, were recruited between January 2023 and December 2024. OA severity was assessed using the Kellgren-Lawrence grading system, and clinical symptoms were assessed using the WOMAC score. Statistical analyses included group comparisons, Pearson correlations with Benjamini-Hochberg FDR adjustment, ROC curves for diagnostic performance, and multivariate logistic regression to identify independent risk factors.

**Results:**

Among patients with knee OA, those with Kellgren-Lawrence (K-L) grade III–IV had significantly higher EphA2 levels than those with K-L grades I–II. Receiver operating characteristic (ROC) analysis determined an optimal EphA2 cut-off of 276.8 pg./mL, yielding 92% sensitivity, 72% specificity, and an AUC of 0.924. After false discovery rate (FDR) correction, EphA2 remained positively correlated with the WOMAC score (*r* = 0.363, *q* < 0.003), ESR (*r* = 0.251, *q* < 0.006), TNF-*α* (*r* = 0.213, *q* < 0.012), MDA (*r* = 0.238, *q* < 0.009), COMP (*r* = 0.208, *q* < 0.018), MMP-13 (*r* = 0.200, *q* < 0.021), IL-6 (*r* = 0.198, *q* < 0.024), and ACSL4 (*r* = 0.200, *q* < 0.021). Consistently, serum EphA2 levels showed strong associations with cartilage degradation markers (COMP, HA, MMP-13, and CTX-2), inflammatory cytokines (TNF-α, IL-1β, IL-6, and IL-17A), oxidative stress (MDA), and ferroptosis (ACSL4), while displaying negative correlations with cartilage synthesis markers (PIICP and aggrecan) and antioxidant defenses (GSH and GPX4). Multivariate logistic regression further identified EphA2 (OR = 1.019, 95% CI: 1.009–1.029, *p* < 0.001), WOMAC, and TNF-α as independent risk factors for poor prognosis in knee OA.

**Conclusion:**

These findings suggest that EphA2 is closely associated with cartilage degradation, inflammation, oxidative stress, and ferroptosis in knee OA and may serve as a promising biomarker for disease diagnosis and progression monitoring.

## Introduction

Osteoarthritis (OA) is the most common degenerative joint disease, affecting over 500 million people worldwide and representing a leading cause of disability in the elderly population (1). The disease is characterized by progressive articular cartilage degradation, subchondral bone remodeling, osteophyte formation, and synovial inflammation, ultimately leading to joint pain, stiffness, and functional impairment (2). Despite its high prevalence and socioeconomic burden, there are currently no curative treatments, and diagnosis largely relies on radiographic evaluation and clinical symptoms, which often detect the disease only at advanced stages.

Inflammation plays a crucial role in the pathophysiology of OA. Pro-inflammatory cytokines, such as interleukin-1β (IL-1β), tumor necrosis factor-alpha (TNF-*α*), and interleukin-6 (IL-6), are elevated in OA-affected joints and synovial fluid (3). These cytokines not only stimulate chondrocyte catabolism but also activate synovial fibroblasts, thereby amplifying local inflammation. Importantly, they promote the expression and activation of matrix-degrading enzymes, notably matrix metalloproteinases (MMPs) and aggrecanases (ADAMTS family), which mediate the irreversible breakdown of the cartilage extracellular matrix (4, 5). Consequently, the simultaneous measurement of cytokine concentrations and enzyme activity levels provides valuable insights into both the inflammatory status and catabolic burden within the OA joint environment.

The identification of reliable, minimally invasive biomarkers that reflect early joint degeneration and correlate with disease severity is a major unmet need in OA research (6). Several serum and synovial fluid markers, such as cartilage oligomeric matrix protein (COMP), C-terminal cross-linked telopeptide of type II collagen (CTX-II), and MMPs, have been explored; however, their diagnostic and prognostic utility remains limited owing to their variable specificity and inconsistent association with disease progression (7, 8).

EphA2, a member of the ephrin receptor tyrosine kinase family, is widely recognized for its role in embryonic development, angiogenesis, and tumorigenesis (9). Emerging evidence suggests that EphA2 also plays a role in skeletal and joint biology, influencing bone remodeling by regulating osteoclast and osteoblast differentiation (10). Recent transcriptomic and experimental studies have revealed that EphA2 expression is markedly upregulated in osteoarthritic cartilage and synovium, promoting pro-inflammatory signaling, chondrocyte hypertrophy, and extracellular matrix degradation via MAPK and NF-κB pathways (11). Furthermore, pharmacologic inhibition of EphA2 in OA models has been shown to attenuate cartilage degeneration and reduce the expression of catabolic enzymes, such as MMP-13 (11).

EphA2 has been extensively studied in oncology, where it regulates tumor growth, angiogenesis, and metastasis (12, 13). Emerging evidence suggests that EphA2 may also contribute to OA pathology by promoting chondrocyte apoptosis, extracellular matrix degradation, and inflammatory responses (14). Although overexpression of EphA2 in joint tissues has been reported, its systemic expression and relationship with disease severity remain unclear, particularly in musculoskeletal disorders, such as knee OA. Preclinical studies further support targeting EphA2 in OA, as it is selectively upregulated in hypertrophic chondrocytes compared to other Eph receptors. *In vitro* inhibition attenuates the expression of inflammatory mediators and hypertrophic differentiation, whereas *in vivo* blockade with ALW-II-41-27 reduces synovial inflammation and cartilage damage (11), highlighting its potential as a promising biomarker. Therefore, investigating serum EphA2 levels may provide novel insights into its clinical significance, serving as a convenient biomarker for disease activity and progression. Accordingly, the present study aimed to evaluate serum EphA2 levels in patients with knee OA compared to those in healthy controls and examine their association with radiographic severity and clinical parameters.

## Materials and methods

### Subjects

**Figure 1:**
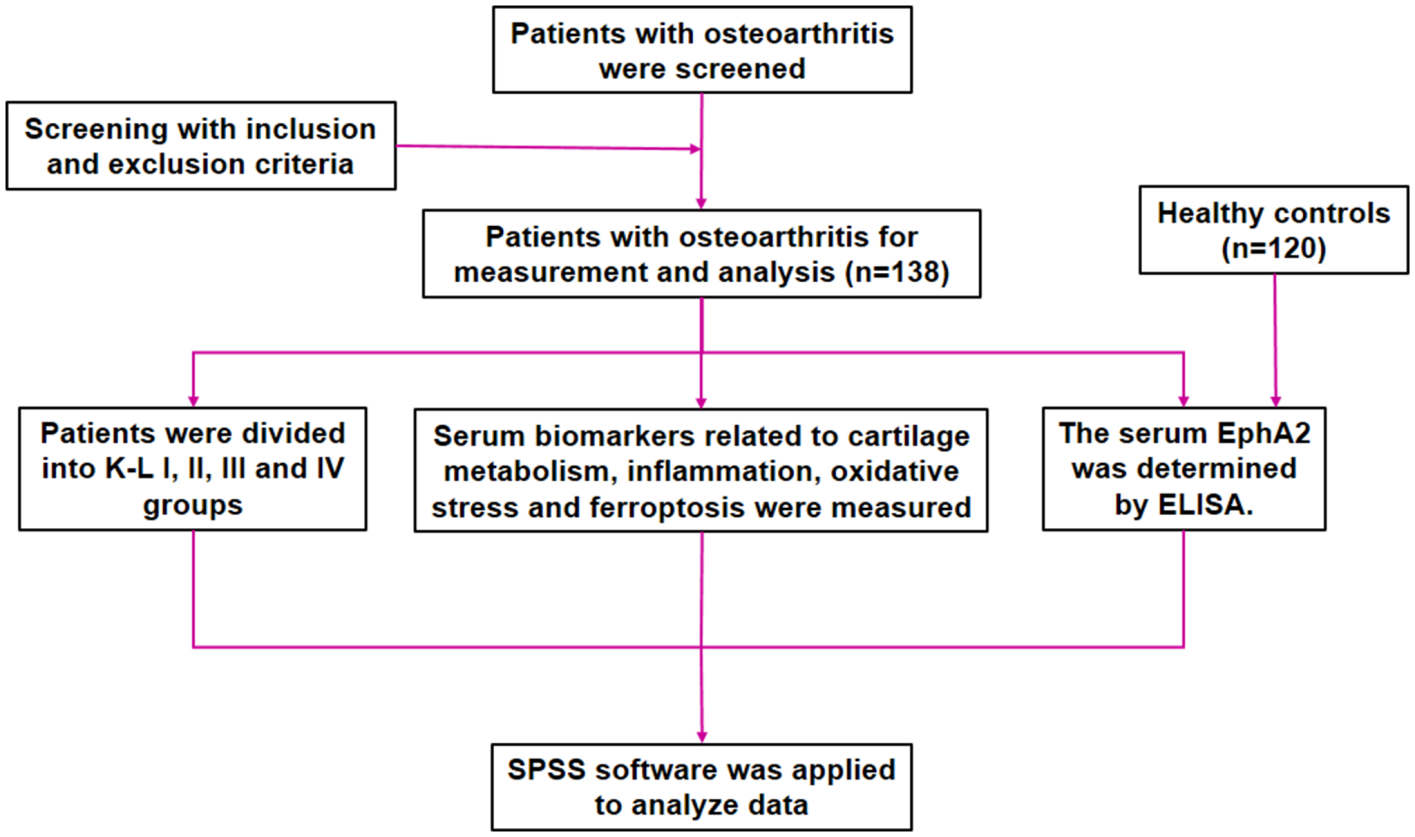
Flow chart of patient screening, selection, measurement, and analysis.

A total of 258 participants (138 patients with knee OA and 120 healthy controls) were included from the Department of Orthopedics in our Hospital between January 2023 and December 2024. Healthy controls were selected from subjects who underwent physical examinations and had matched age and sex. All patients with knee OA were diagnosed according to the criteria of the American College of Rheumatology (15). The inclusion criteria were as follows: (i) all patients were diagnosed with primary knee OA; (ii) all patients were >18 years old; (iii) all patients had radiological evidence of OA, and were divided according to the Kellgren-Lawrence (K-L) score of I-IV. There were 38, 43, 35, and 22 cases of grades I, II, III, and IV, respectively, in this study. Extensive exclusion criteria were applied to reduce potential confounding factors that could affect the study results. The exclusion criteria were as follows: (i) clinically evident OA affecting other joints (e.g., hip); (ii) secondary OA or rheumatoid arthritis; (iii) coexisting autoimmune disorders, systemic inflammatory conditions, severe infections, malignant neoplasms, or significant cardiac, hepatic, or renal impairment; (iv) receipt of intra-articular injections within the preceding week, or immunotherapy or anti-inflammatory therapy within the preceding 3 months; and (v) a documented history of knee injury or surgical intervention. This study complied with the Declaration of Helsinki and was approved by the hospital ethics committee. Participant recruitment was concluded on December 31, 2024; all biomarker assays and statistical analyses were completed during the first half of 2025, once the data were locked. Figure 1 illustrates an overview of the study’s framework.

### Sample size

The required sample size was estimated using the MedSci APP with the formula *n* = 2(Z_*α*/2_ + Z_β_)^2^ *σ*^2^/δ^2^, where Z_α/2_ = 1.96 (α = 0.05), Z_β_ = 1.28 (90% power), σ is the pooled standard deviation, and 𝛿 is the expected mean difference. Based on the mean serum EphA2 levels in controls (258.4 ± 40.4) and patients with OA (354.3 ± 52.9), the minimum sample size was 4 per group. Our study included 120 controls and 138 patients with OA, which exceeded this requirement.

### Assessment of severity in patients with OA

All patients with knee OA underwent X-ray examination and were classified according to the Kellgren-Lawrence (K-L) grading standard (16): Grade 0 indicates no change in imaging; Grade I indicates normal joint space but with bone spur-like osteophytes; Grade II is characterized by mild narrowing of the joint space and the appearance of obvious bone spurs like osteophytes; Grade III is characterized by narrow gaps in the joints, obvious osteophyte formation, abnormal joint contours, and bone sclerosis; and Grade IV is characterized by significant narrowing of the joint space, appearance of large osteophytes, deformity of the joint contour, and severe bone sclerosis. Knee OA is defined as at least one knee joint with K-L grade> I. When both knees of the patient met the K-L grade, the higher grade was adopted.

### Assessment of disease severity

The clinical symptom assessment of knee OA adopts the Western Ontario and McMaster Universities Osteoarthritis Index (WOMAC) score. The clinical symptoms of the patients were evaluated, mainly including four dimensions (pain, morning stiffness, function, and quality of life), with a total score of 88 points. Higher scores indicate more severe symptoms.

### Data collection

The age, sex, and body mass index (BMI) of all participants were collected. All fasting blood samples were collected between 8:00 a.m. and 10:00 a.m. following an overnight fast of at least 8 h, following standard clinical research practice (17). Erythrocyte sedimentation rate (ESR) was measured using an automatic biochemical analyzer.

### Knee function

The joint function of patients was evaluated using the Lysholm knee score, which includes limping, support, locking, instability, pain, swelling, climbing stairs, and squatting. Higher scores indicate better knee function.

### Follow up and grouping

Patients with OA were followed up for 6 months. At the last follow-up, patients with Lysholm knee function scores ≥70 points were regarded as the good prognosis group, and those with Lysholm knee function scores <70 points were regarded as the poor prognosis group.

### Measurement of serum biomarkers

The blood samples were collected from 138 patients with knee OA and 120 healthy controls. Blood was drawn into serum separation tubes (SSTs) containing a clot activator, allowed to clot at room temperature, and centrifuged at 3,000 rpm for 10 min to separate the serum. The resulting serum was aliquoted and stored at −80 °C until analysis to preserve biomarker stability (18). Serum EphA2 (ml038095, Shanghai Enzyme-linked Biotechnology, Shanghai, China), COMP (DCMP0, R&D Systems), Hyaluronan (HA) (DHYAL0, R&D Systems), MMP-13 (SEKH-0259, Solarbio, China), CTX-2 (ml105642, Shanghai Enzyme-linked Biotechnology, Shanghai, China), PIICP (AB5126, EK-Bioscience), aggrecan (DY1220, R&D Systems), TNF-*α* (DTA00D, R&D Systems), IL-1β (DLB50, R&D Systems), IL-6 (D6050B, R&D Systems), IL-17A (D1700, R&D Systems), MDA (EU2577, FineTest), GSH (ml038257, Shanghai Enzyme-linked Biotechnology), GPX4 (ml060706, Shanghai Enzyme-linked Biotechnology), and ACSL4 (EH6088, FineTest) levels were measured using an ELISA kit, and absorbance at 450 nm was assessed using a microplate reader and each experiment run in duplicate (19). All assays were performed by laboratory personnel who were blinded to the participants’ clinical status and K-L grade.

### Statistical analysis

Statistical analyses were performed using SPSS version 20.0. Continuous variables are presented as mean ± standard deviation (SD). The Kolmogorov–Smirnov test was used to assess normality. Normally distributed data were compared using an unpaired Student’s *t*-test, while non-normally distributed data were analyzed using the Kruskal–Wallis H test. Categorical variables are expressed as frequencies (percentages) and were compared using the chi-square test. The Mann–Whitney U test was used for quantitative comparisons between the K-L I–II and K-L III–IV groups. Pearson’s correlation analysis was used to evaluate the relationship between EphA2 expression and other continuous variables. To account for multiple comparisons, *p*-values for correlation analyses were adjusted using the Benjamini–Hochberg false discovery rate (FDR) procedure, with a *q* < 0.05 considered statistically significant. Receiver operating characteristic (ROC) curves were generated to assess the diagnostic value of EphA2 in knee OA. The independent factors influencing EphA2 expression were determined using multivariate logistic regression analysis. A two-sided *p* < 0.05 was considered statistically significant.

## Results

### Demographic characteristics

This study employed a comprehensive approach to compare patients with knee OA to healthy controls. Student’s *t*-test analysis showed that individuals with knee OA had significantly higher levels of several clinical and biochemical indicators, including the body mass index (BMI), erythrocyte sedimentation rate (ESR), cartilage oligomeric protein (COMP), hyaluronic acid (HA), matrix metalloproteinase-13 (MMP-13), C-terminal telopeptide of type II collagen (CTX-2), tumor necrosis factor-alpha (TNF-*α*), interleukin-1 beta (IL-1β), interleukin-6 (IL-6),interleukin-17A (IL-17A), malondialdehyde (MDA), acyl-CoA synthetase family 4 (ACSL4), and ephrin type-A receptor 2 (EphA2) than healthy controls (Table 1). Conversely, patients with knee OA exhibited significantly lower levels of procollagen II C-terminal propeptide (PIICP), aggrecan, glutathione (GSH), and glutathione peroxidase 4 (GPX4). No significant differences were observed between the groups in terms of age or sex (*p* > 0.05).

**Table 1:** Baseline characteristics of all subjects.

Characteristics	Control (*n* = 120)	Knee osteoarthritis (*n* = 138)	*P*-value
Age (years)	55.87 ± 7.56	55.83 ± 6.60	0.963
Sex (male, %)	56 (46.7%)	62 (44.9%)	0.780
BMI (kg/m^2^)	24.04 ± 2.79	24.75 ± 2.77	0.040
ESR (mm/h)	8.59 ± 1.14	16.59 ± 2.88	<0.001
COMP (ng/mL)	241.21 ± 36.67	383.03 ± 59.62	<0.001
HA (ng/mL)	80.22 ± 16.77	119.78 ± 27.95	<0.001
MMP-13 (ng/mL)	14.83 ± 2.36	22.95 ± 4.16	<0.001
CTX-2 (pg/mL)	406.29 ± 53.70	567.77 ± 83.72	<0.001
PIICP (pg/mL)	660.43 ± 85.12	449.45 ± 67.06	<0.001
Aggrecan (ng/mL)	279.83 ± 46.16	217.10 ± 41.63	<0.001
TNF-α (pg/mL)	40.90 ± 5.09	66.19 ± 9.14	<0.001
IL-1β (pg/mL)	13.18 ± 1.62	25.96 ± 3.23	<0.001
IL-6 (pg/mL)	47.64 ± 6.69	74.92 ± 12.05	<0.001
IL-17A (pg/mL)	31.01 ± 3.67	46.91 ± 5.65	<0.001
MDA (nmol/mL)	7.07 ± 1.11	8.97 ± 1.45	<0.001
GSH (nmol/mL)	9.62 ± 1.32	7.26 ± 1.03	<0.001
GPX4 (ng/mL)	173.22 ± 27.73	136.66 ± 22.26	<0.001
ACSL4 (pg/mL)	1.21 ± 0.28	1.50 ± 0.35	<0.001
EphA2 (pg/mL)	258.46 ± 40.41	354.30 ± 52.90	<0.001

Continuous variables are expressed as mean ± SD, and analyzed using t test or Wilcoxon-Mann–Whitney test. Categorical variables are expressed as frequency (percentage), and analyzed using the chi-square test. BMI, body mass index; ESR, erythrocyte sedimentation rate; COMP, cartilage oligomeric protein; HA, hyaluronic acid; MMP-13, matrix metalloproteinase-13; CTX-2, C-terminal telopeptide of type II collagen; PIICP, procollagen II C-terminal propeptide; TNF-α, tumor necrosis factor-alpha; IL-1β, interleukin-1 beta; IL-6, interleukin-6; IL-17A, interleukin-17A, MDA, malondialdehyde; GSH, glutathione; GPX4, glutathione peroxidase 4; ACSL4, acyl-CoA synthetase family 4; EphA2, ephrin type-A receptor 2.

Furthermore, we performed the Mann–Whitney U test to compare K-L grades I-II (*n* = 81) and K-L grades III-IV (*n* = 57) in patients with knee OA (*n* = 138) based on the Kellgren-Lawrence (KL) score. The results showed that K-L III-IV knee OA patients showed significantly elevated levels of different health indicators, including the Western Ontario and McMaster Universities Osteoarthritis Index (WOMAC), ESR, COMP, HA, MMP-13, CTX-2, TNF-*α*, IL-1β, IL-6, IL-17A, MDA, ACSL4, and EphA2, than K-L I-II (Table 2). In contrast, K-L I-II had lower levels of markers such as PIICP, aggrecan, GSH, and GPX4, than healthy controls. However, there were no significant differences in age, sex, or BMI between K-L grades I-II and III-IV (*p* > 0.05).

**Table 2:** Baseline characteristics of all patients with osteoarthritis grouped by Kellgren-Lawrence (KL) score.

Characteristics	K-L I-II (*n* = 81)	K-L III-IV (*n* = 57)	*P*-value
Age (years)	55.49 ± 6.96	56.30 ± 6.07	0.560
Sex (male, %)	37 (45.7%)	25 (43.9%)	0.832
BMI (kg/m^2^)	24.48 ± 2.62	25.13 ± 2.95	0.204
WOMAC	35.02 ± 2.62	46.32 ± 4.46	<0.001
ESR (mm/h)	15.87 ± 2.60	17.58 ± 2.97	0.001
COMP (ng/mL)	366.53 ± 54.81	406.47 ± 58.74	<0.001
HA (ng/mL)	115.62 ± 25.85	125.71 ± 29.92	0.030
MMP-13 (ng/mL)	22.09 ± 3.71	24.18 ± 4.47	0.028
CTX-2 (pg/mL)	550.88 ± 79.21	591.77 ± 84.76	0.005
PIICP (pg/mL)	460.04 ± 68.28	434.39 ± 62.84	0.050
Aggrecan (ng/mL)	226.19 ± 42.11	204.19 ± 37.65	0.004
TNF-α (pg/mL)	64.65 ± 8.74	68.39 ± 9.32	0.012
IL-1β (pg/mL)	24.65 ± 2.81	27.82 ± 2.87	<0.001
IL-6 (pg/mL)	72.26 ± 12.19	78.71 ± 10.87	0.002
IL-17A (pg/mL)	44.87 ± 5.15	49.79 ± 5.08	<0.001
MDA (nmol/mL)	8.44 ± 1.14	9.72 ± 1.51	<0.001
GSH (nmol/mL)	7.63 ± 0.96	6.73 ± 0.89	<0.001
GPX4 (ng/mL)	140.77 ± 23.28	130.83 ± 19.46	0.021
ACSL4 (pg/mL)	1.41 ± 0.33	1.62 ± 0.34	<0.001
EphA2 (pg/mL)	338.74 ± 45.25	376.41 ± 55.44	<0.001

Continuous variables are expressed as mean ± SD, and analyzed using Mann–Whitney U test. Categorical variables are expressed as frequency (percentage), and analyzed using the chi-square test. BMI, body mass index; WOMAC, Western Ontario and McMaster Universities Osteoarthritis Index; ESR, erythrocyte sedimentation rate; COMP, cartilage oligomeric protein; HA, hyaluronic acid; MMP-13, matrix metalloproteinase-13; CTX-2, C-terminal telopeptide of type II collagen; PIICP, procollagen II C-terminal propeptide; TNF-α, tumor necrosis factor-alpha; IL-1β, interleukin-1 beta; IL-6, interleukin-6; IL-17A, interleukin-17A, MDA, malondialdehyde; GSH, glutathione; GPX4, glutathione peroxidase 4; ACSL4, acyl-CoA synthetase family 4; EphA2, ephrin type-A receptor 2.

### Serum EphA2 levels are higher in patients with knee OA

**Figure 2:**
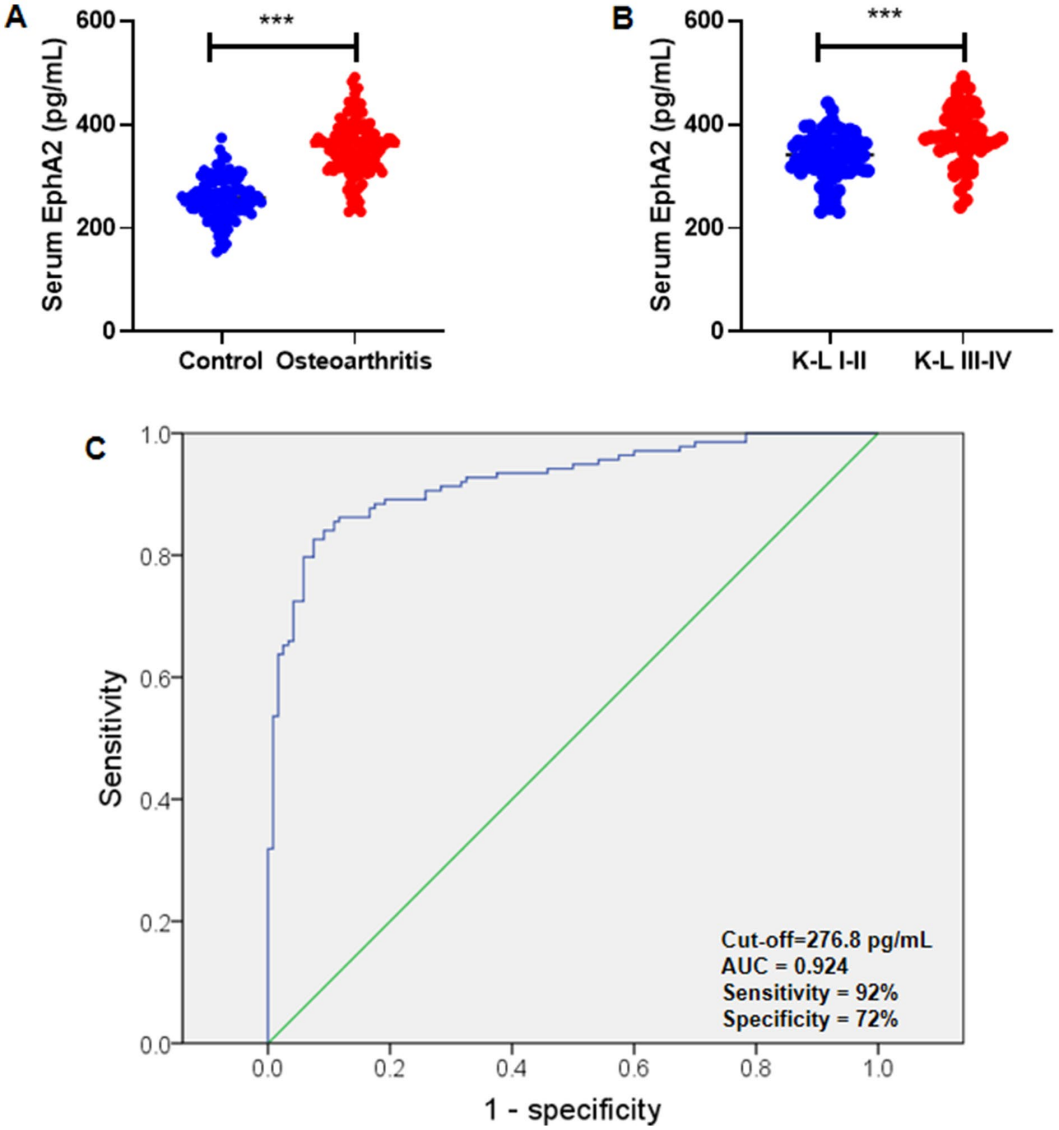
Serum EphA2 were measured in healthy controls and osteoarthritis patients. **(A)** Serum EphA2 levels were detected by ELISA and compared between healthy controls and patients with osteoarthritis. The serum EphA2 concentration in patients with osteoarthritis (*n* = 138) was significantly higher than that in healthy controls (*n* = 120). **(B)** Serum EphA2 levels were compared between patients with osteoarthritis with K-L scores I-II and K-L scores III-IV. The serum EphA2 concentration in patients with OA with K-L scores III-IV (*n* = 57) was significantly higher than that in patients with OA with K-L scores I-II (*n* = 81). **(C)** The ROC curve was used to obtain the critical point of serum EphA2 levels that distinguished between patients with osteoarthritis and healthy controls. The optimal critical point was 276.8 pg./mL. A *t*-test was conducted, with significance indicated as ***p < 0.001.

ELISA was used to measure serum levels of EphA2 in patients with knee OA (*n* = 138) and healthy controls (*n* = 120). We further divided all patients with knee OA into those with K-L scores of I-II (*n* = 57) and those with K-L scores of III-IV (*n* = 81). We found that the serum EphA2 concentration in patients with knee OA was significantly higher than that in the healthy controls (Figure 2A). We also observed that the serum EphA2 concentration in patients with knee OA with K-L scores of III-IV was considerably higher than that in patients with knee OA with K-L scores of I-II (Figure 2B). Additionally, Receiver Operating Characteristic (ROC) curve analysis indicated that the optimal cutoff value for serum EphA2 was 276.8 pg./mL, with a sensitivity of 92% and specificity of 72%, resulting in an Area Under the Curve (AUC) of 0.924 (Figure 2C).

### Correlation between serum EphA2 and clinical indicators

After adjusting for multiple comparisons using the Benjamini–Hochberg false discovery rate (FDR) procedure, serum EphA2 levels remained significantly positively correlated with the WOMAC score (*r* = 0.363, *q* < 0.003), ESR (*r* = 0.251, *q* < 0.006), TNF-*α* (*r* = 0.213, *q* < 0.012), MDA (*r* = 0.238, *q* < 0.009), COMP (*r* = 0.208, *q* < 0.018), MMP-13 (*r* = 0.200, *q* < 0.021), IL-6 (*r* = 0.198, *q* < 0.024), and ACSL4 (*r* = 0.200, *q* < 0.021) (Table 3). Correlations with other parameters were not significant after FDR correction, indicating that EphA2 is robustly associated with key indicators of clinical severity, inflammation, oxidative stress, and cartilage metabolism in knee OA.

**Table 3:** Correlation of serum EphA2 with clinical and biochemical parameters in patients with knee OA (*n* = 138).

Parameters	EphA2 (*n* = 138)			
	*r*	*P*-value	Sort	Corrected P cut-off (i/m × α)
Age (years)	0.051	0.550	17	0.05
BMI (kg/m^2^)	0.101	0.237	16	0.0471
WOMAC	0.363	<0.001	1	0.00294
ESR (mm/h)	0.251	0.003	2	0.00588
COMP (ng/mL)	0.208	0.014	6	0.0176
HA (ng/mL)	0.190	0.026	10	0.0294
MMP-13 (ng/mL)	0.200	0.019	7	0.0206
CTX-2 (pg/mL)	0.183	0.031	13	0.0382
PIICP (pg/mL)	−0.188	0.027	11	0.0324
Aggrecan (ng/mL)	−0.178	0.036	14	0.0412
TNF-α (pg/mL)	0.213	0.012	4	0.0117
IL-1β (pg/mL)	0.191	0.025	9	0.0265
IL-6 (pg/mL)	0.198	0.020	8	0.0235
IL-17A (pg/mL)	0.179	0.036	15	0.0441
MDA (nmol/mL)	0.238	0.005	3	0.00882
GSH (nmol/mL)	−0.210	0.013	5	0.0147
GPX4 (ng/mL)	−0.187	0.028	12	0.0353
ACSL4 (pg/mL)	0.200	0.019	7	0.0206

The correlation between serum EphA2 and clinical indicators was analyzed by Pearson’s correlation test. With 18 biomarkers, we performed Benjamini–Hochberg for false-discovery rate correction. When p value < Corrected P cut-off, the correlation between the biomarker and EphA2 is significant. i, sequence number sorted from small to large; *m* = 17; α = 0.05.

**Figure 3:**
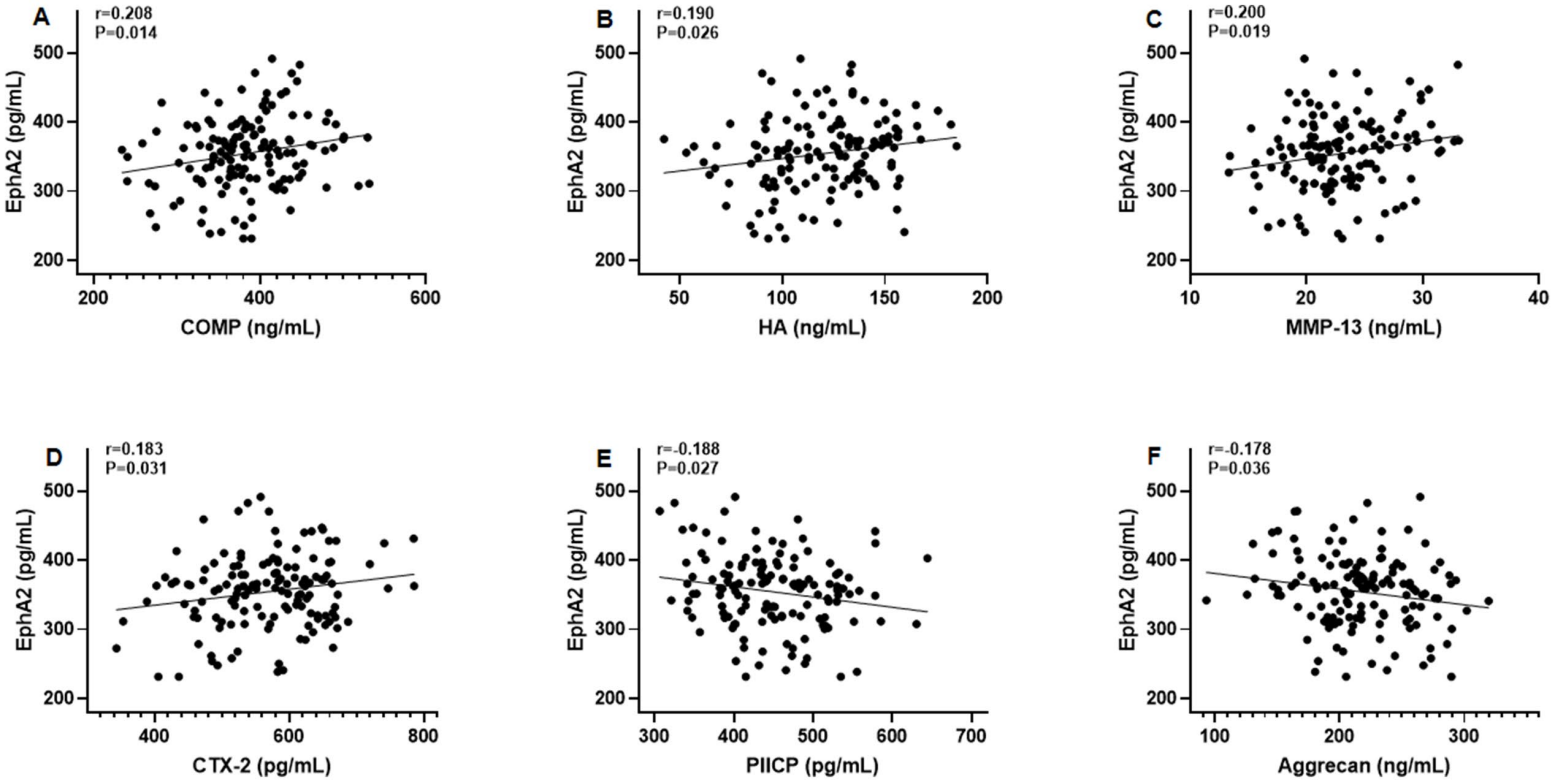
Correlation between serum EphA2 levels and serum indicators of cartilage metabolism. Pearson correlation test was performed between EphA2 and **(A)** COMP, **(B)** Hyaluronan (HA), **(C)** MMP-13, **(D)** CTX-2, **(E)** PIICP, and **(F)** aggrecan in all osteoarthritis patients.

**Figure 4:**
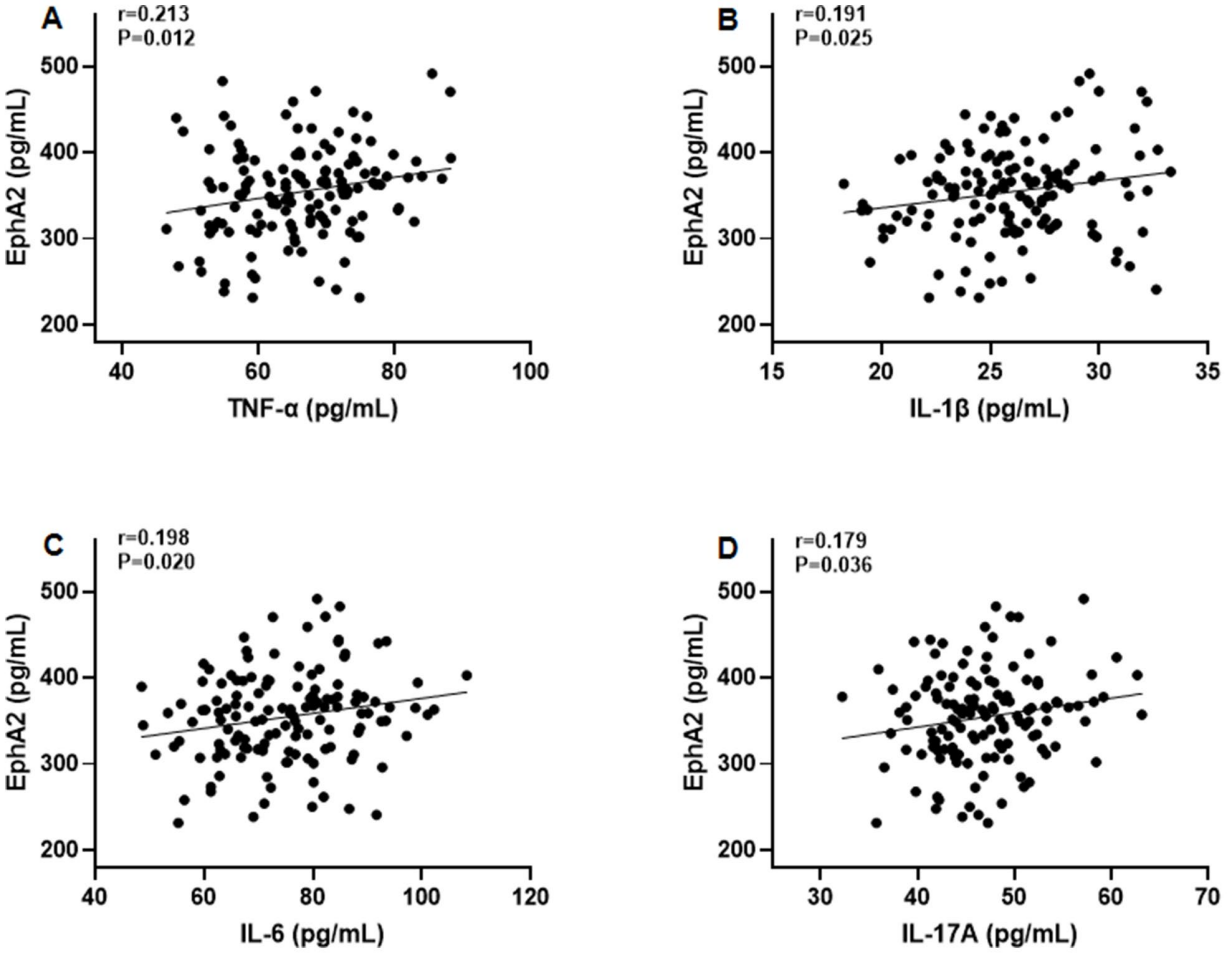
The correlation between serum EphA2 levels and serum inflammatory cytokines. Pearson correlation test was performed between EphA2 and **(A)** TNF-*α*, **(B)** IL-1β, **(C)** IL-6, and **(D)** IL-17A in all patients with osteoarthritis.

**Figure 5:**
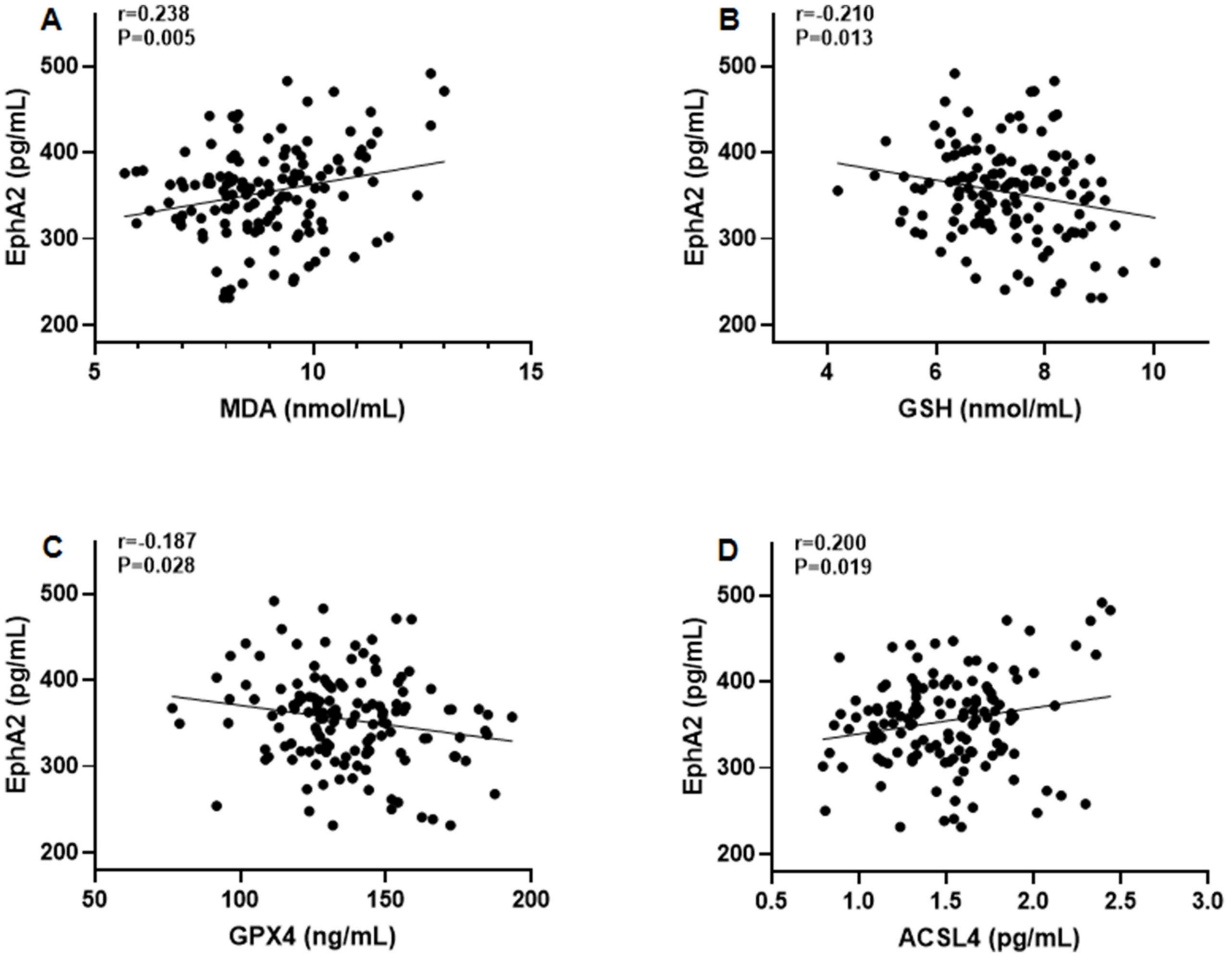
The correlation between serum EphA2 levels and serum indicators of oxidative stress and ferroptosis. Pearson correlation test was performed between EphA2 and **(A)** MDA **(B)** GSH **(C)** GPX4, and **(D)** ACSL4 in all patients with osteoarthritis.

Furthermore, Pearson’s correlation test revealed that serum EphA2 levels were positively correlated with serum indicators of cartilage metabolism, including COMP (*r* = 0.208, *p* = 0.014), HA (*r* = 0.190, *p* = 0.026), MMP-13 (*r* = 0.200, *p* = 0.019), and CTX-2 (*r* = 0.183, *p* = 0.031), and negatively correlated with PIICP (*r* = −0.188, *p* = 0.027), and aggrecan (*r* = −0.178, *p* = 0.036), in all patients with knee OA (Figure 3). In addition, we observed that serum EphA2 levels were positively correlated with serum levels of inflammatory cytokines such as TNF-α (*r* = 0.213, *p* = 0.012), IL-1β (*r* = 0.191, *p* = 0.025), IL-6 (*r* = 0.198, *p* = 0.020), and IL-17A (*r* = 0.179, *p* = 0.036) (Figure 4). Furthermore, we found that serum EphA2 levels were positively correlated with indicators of oxidative stress and ferroptosis, including MDA (*r* = 0.238, *p* = 0.005) and ACSL4 (*r* = 0.200, *p* = 0.019), and negatively associated with GSH (*r* = −0.210, *p* = 0.013) and GPX4 (*r* = −0.187, *p* = 0.028) (Figure 5). Finally, multivariate logistic regression identified WOMAC (OR = 1.074, 95% CI: 1.005–1.147, *p* = 0.034), TNF-*α* (OR = 1.062, 95% CI: 1.010–1.114, *p* = 0.018), and EphA2 (OR = 1.019, 95% CI: 1.009–1.029, *p* < 0.001) as independent risk factors for poor prognosis in patients with OA (Table 4).

**Table 4:** Logistic multivariate regression for poor prognosis of OA patients.

Characteristics	Odds ratio	95% confidence interval	*P*-value
WOMAC	1.074	1.005–1.147	0.034
TNF-α (pg/mL)	1.062	1.010–1.114	0.018
EphA2 (pg/mL)	1.019	1.009–1.029	<0.001

WOMAC, Western Ontario and McMaster Universities Osteoarthritis Index; TNF-α, tumor necrosis factor-alpha; EphA2, ephrin type-A receptor 2.

## Discussion

In this study, we comprehensively evaluated the biochemical, inflammatory, oxidative stress, and ferroptosis-related profiles of patients with knee OA and compared them to those of healthy controls. Our findings confirmed that knee OA patients exhibited significantly elevated serum levels of cartilage degradation markers (COMP, HA, MMP-13, and CTX-2), pro-inflammatory cytokines (TNF-*α*, IL-1β, IL-6, and IL-17A), oxidative stress markers (MDA), ferroptosis-associated proteins (ACSL4), and notably, EphA2. Conversely, protective cartilage synthesis markers (PIICP and aggrecan) and antioxidant defenses (GSH and GPX4) were markedly reduced. These biochemical alterations were observed despite no significant differences in age or sex between the knee OA and control groups, consistent with prior reports that OA is largely driven by pathological rather than demographic factors (1).

One of the most notable results is the significant elevation of serum EphA2 in knee OA patients compared with healthy controls, with further increases in advanced disease stages (K-L III-IV). ROC analysis demonstrated excellent diagnostic accuracy (AUC = 0.924), exceeding that reported for many conventional OA biomarkers such as COMP (AUC ~ 0.80) (20) and CTX-2 (AUC ~ 0.88) (21). EphA2, a receptor tyrosine kinase, has been well-studied in cancer biology because of its role in cell proliferation, migration, angiogenesis, and resistance to therapy (22). More recently, evidence has implicated it in inflammatory joint disease. Recent transcriptomic and experimental work revealed that EphA2 is upregulated in osteoarthritic cartilage, and that pharmacologic inhibition of EphA2 reduces inflammation, hypertrophic marker expression, and MMP-mediated matrix breakdown in human OA chondrocytes and mouse OA models. The MAPK and NF-κB signaling pathways, both downstream of EphA2 activation, are well-established drivers of cartilage degradation (11, 23). Our results extend these findings to the clinical setting, suggesting that EphA2 may be both a marker and potential driver of OA pathophysiology.

We observed significant positive correlations between EphA2 and established cartilage breakdown markers, including COMP, HA, MMP-13, and CTX-2, and negative correlations with PIICP and aggrecan levels. COMP and CTX-2 are recognized indicators of cartilage degradation and predictors of OA progression (24). MMP-13, the primary collagenase responsible for type II collagen degradation in cartilage, is a critical mediator of OA pathogenesis (25). The association between EphA2 and MMP-13 is particularly noteworthy, as EphA2 has been shown to modulate MMP expression in chondrocyte cultures (26), supporting the idea that EphA2 may directly facilitate extracellular matrix breakdown.

We also found that serum EphA2 levels were positively correlated with inflammatory cytokines, including TNF-*α*, IL-1β, IL-6, and IL-17A, all of which are known to exacerbate cartilage catabolism and synovial inflammation (27). TNF-α and IL-1β stimulate MMP-13 production, whereas IL-6 and IL-17A synergistically enhance inflammatory cartilage destruction (28). The observed relationship between EphA2 and these cytokines is consistent with reports that EphA2 activation amplifies NF-κB– and MAPK-mediated inflammatory signaling in endothelial and immune cells (29), supporting the hypothesis that EphA2 contributes to the chronic inflammatory milieu in OA.

A novel and clinically relevant aspect of this study is the association between EphA2 and oxidative stress and ferroptosis-related markers. We found positive correlations between EphA2, MDA and ACSL4, along with negative correlations between EphA2, GSH and GPX4. Oxidative stress has long been implicated in OA, driving chondrocyte apoptosis and matrix degradation (30). Ferroptosis, an iron-dependent cell death process characterized by lipid peroxidation, has recently been recognized as a critical contributor to cartilage degeneration (31). ACSL4 promotes the esterification of polyunsaturated fatty acids, generating substrates for lipid peroxidation, whereas GPX4 and GSH serve as key inhibitors of ferroptosis by reducing the levels of lipid hydroperoxides. Our results suggest that EphA2 may be involved in ferroptotic signaling pathways in OA, a hypothesis that warrants further research.

Importantly, although we observed parallel associations between EphA2 and ferroptosis markers, the potential mechanistic link requires deeper consideration. EphA2 activates the MAPK/ERK and PI3K/Akt signaling cascades (22, 23), which are also implicated in the regulation of ferroptosis sensitivity (32, 33). For example, ERK activation enhances ACSL4 expression and lipid peroxidation, thereby promoting ferroptosis (34). Conversely, the PI3K/Akt/Nrf2 axis upregulates GPX4 and GSH biosynthesis, thereby suppressing ferroptosis (35). Dysregulated EphA2 signaling in OA chondrocytes may tilt the balance toward ERK-driven ACSL4 upregulation and impaired Nrf2-mediated antioxidant defense, favoring ferroptotic cell death. This hypothesis is consistent with the correlations observed in this study and provides a testable mechanistic framework for future studies.

Previous OA biomarker studies have primarily focused on individual catabolic or inflammatory markers, with limited integration of molecular pathways. For instance, CTX-2 and COMP predict OA progression but do not capture the full inflammatory or oxidative profile (20, 21). By integrating cartilage metabolism (MMP-13 and CTX-2), inflammation (TNF-α, IL-1β, IL-6, and IL-17A), oxidative stress (MDA), and ferroptosis markers (ACSL4 and GPX4) with EphA2, our study highlights EphA2 as a central node reflecting multiple aspects of OA pathology. This multi-pathway association distinguishes EphA2 from traditional biomarkers with a single mechanism.

Our cohort’s relatively young age and low BMI, despite a substantial proportion of patients with advanced radiographic OA-are consistent with patterns observed in populations engaged in high physical activity or labor-intensive occupations, where chronic mechanical joint loading may contribute to OA development independently of obesity-related risk factors (36). BMI is a crude measure of adiposity and does not account for body composition; in lean individuals with OA, reduced muscle mass and increased joint stress may play more important roles in disease progression (37, 38). Furthermore, inflammatory biomarkers such as cytokines and ESR in our cohort were generally low and within the reference ranges. While some between-group differences reached statistical significance, these changes are unlikely to have substantial clinical relevance in early or moderate OA, as low-grade inflammation in OA often does not produce marked alterations in systemic inflammatory markers (39, 40).

## Limitations

This study had several limitations. First, our study spanned a 2-year period (January 2023–December 2024) and employed a cross-sectional design, which limits the ability to infer causality; thus, our findings should be interpreted as associations rather than as causal relationships. Future longitudinal studies with extended follow-up are warranted to determine the predictive value of EphA2 and elucidate its underlying mechanisms in OA progression. Second, this study did not analyze OA subtypes, which may differ in molecular characteristics. Future studies with larger cohorts and detailed metabolic profiling are warranted to explore whether EphA2 expression varies across OA phenotypes. Third, the younger age distribution and absence of patients over 65 years in our study likely reflect sampling bias from recruiting only in specific outpatient clinics and community settings rather than the general population. Future studies should use population-based sampling, including multiple hospitals, primary care centers, and community sources, to ensure the inclusion of older adults and reduce age-related sampling bias. Fourth, analgesic use was not systematically recorded for statistical adjustment; thus, a small degree of bias in the WOMAC outcomes cannot be ruled out. Future studies should systematically document and adjust for all analgesic uses to more accurately isolate the relationship between biomarkers and clinical status. Fifth, although we adjusted for BMI in the multivariate analyses, residual confounding by unmeasured factors such as medication use, physical activity, and comorbidities cannot be excluded, underscoring the need for future studies with larger cohorts and more comprehensive clinical data to validate our findings. Finally, while our findings suggest a strong association between serum EphA2 levels and OA pathogenesis and disease severity, the underlying mechanisms remain unclear. Future functional studies, including *in vitro* chondrocyte models and *in vivo* OA models, are necessary to determine whether EphA2 acts as a causal driver or merely as a correlated “bystander” biomarker.

## Conclusion

**Figure 6:**
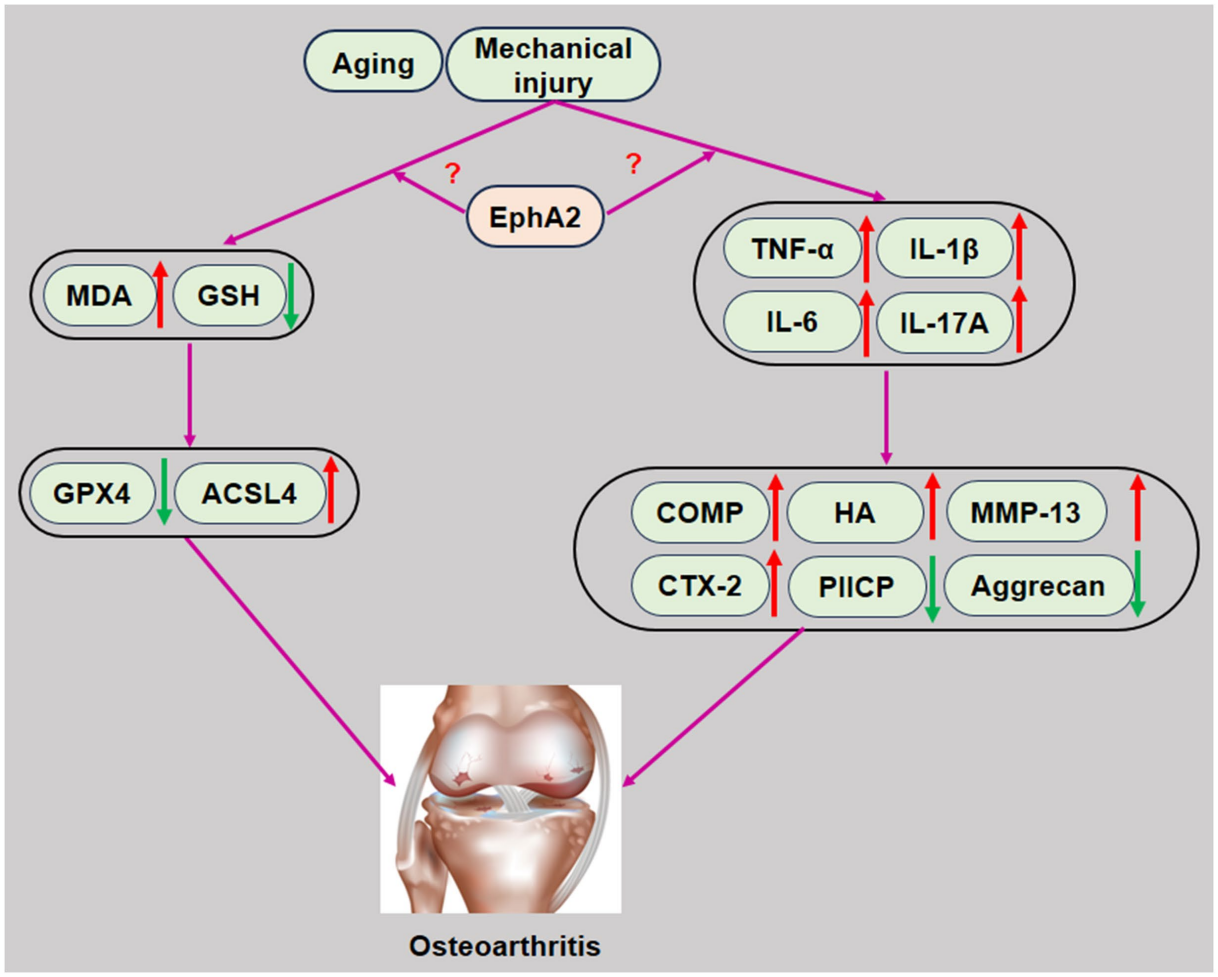
Schematic representation of EphA2 in the development of osteoarthritis. Increased EphA2 levels in patients with osteoarthritis promote chondrocyte injury by promoting inflammation, oxidative stress, and ferroptosis, resulting in abnormal cartilage metabolism. Therefore, increased serum EphA2 levels play an aggravating role in chondrocyte injury in patients with osteoarthritis.

In summary, our study demonstrated that serum EphA2 levels were significantly elevated in patients with knee OA, particularly in those with advanced K-L grades, and exhibited strong diagnostic potential (AUC = 0.924). EphA2 levels were positively correlated with cartilage degradation markers, inflammatory cytokines, and oxidative stress indicators and negatively correlated with anabolic and antioxidant markers (Figure 6). These results suggest that EphA2 is a promising biomarker for the diagnosis of OA and assessment of disease severity. However, as this study was cross-sectional, causal relationships could not be inferred, and further mechanistic studies are required to explore its biological role in OA progression.

## Data Availability

The original contributions presented in the study are included in the article/supplementary material, further inquiries can be directed to the corresponding author.
